# Genome-Wide Identification of *CONSTANS*-*like* (*COL*) Gene Family and the Potential Function of *ApCOL08* Under Salt Stress in *Andrographis paniculata*

**DOI:** 10.3390/ijms26020724

**Published:** 2025-01-16

**Authors:** Yizhu Zhao, Jiahao Xu, Xinyi Xu, Hui Liu, Qinxiang Chang, Ling Xu, Zongsuo Liang

**Affiliations:** 1 Zhejiang Province Key Laboratory of Plant Secondary Metabolism and Regulation, College of Life Sciences and Medicine, Zhejiang Sci-Tech University, Hangzhou 310018, China; 15868190169@163.com (Y.Z.);; 2 The UWA Institute of Agriculture, UWA School of Agriculture and Environment, Faculty of Science, The University of Western Australia, Crawley, WA 6009, Australia

**Keywords:** genome-wide, *Andrographis paniculata*, *CONSTANS*-*like* (*COL*), abiotic stress, expression profile

## Abstract

*Andrographis paniculata* is an important medicinal herb known as a “natural antibiotic”, which has been used in Southeast Asia for thousands of years. The *CONSTANS-like* (*COL*) gene is an important regulatory factor for plant photoperiod flowering and stress response. However, there is currently no detailed research on the *COL* genes of *A. paniculata*. In our study, we performed a genome-wide analysis of *A. paniculata COL* (*ApCOL*) family members using bioinformatics tools and identified nine *ApCOL* genes. Based on phylogenetic analysis, *ApCOL*s were categorized into three groups, with members of the same group having similar structures. Gene duplication events indicated that only one pair of duplicated genes was identified, possibly caused by segmental duplication. In terms of evolutionary relationships, the COL proteins of *A. paniculata* and *Sesamum indicum* were closely related, showing that they are highly similar in the phylogenetic tree. In addition, *ApCOL* genes showed tissue specificity and were specifically highly expressed mainly in leaves and flowers. Based on the cis-regulatory element prediction results, we examined the expression levels of *ApCOL*s under hormone and salt stress, and *ApCOL08* was significantly induced. With subcellular localization results consistent with the prediction, we transformed *ApCOL08* into yeast and showed significant resistance to salt stress. Our study suggests that *ApCOL* genes have important roles in response to abiotic stress and plant development and initially identifies key genes for future molecular regulation studies.

## 1. Introduction

Flowering is a vital part of the plant reproductive process, marking a shift from trophic to reproductive growth [1,2]. Flowering time is regulated by a combination of internal and external factors, such as photoperiod, hormones, environmental changes, and self-development, enabling plants to better adapt to different environmental conditions [3,4]. The *CONSTANS*-*like* (*COL*) gene is a pivotal regulator of the plant’s photoperiodic response by activating the *flowering locus T* (*FT*) transcription and transferring the FT protein from the leaf phloem to the apical meristematic tissue of the stem to promote plant flowering [5,6,7]. COL is a zinc-finger transcription factor characterized by two conserved structural domains, the N-terminal B-box domain and the C-terminal CCT (CO, CO-like, and TOC1) domain [8]. The B-box domain primarily serves as a protein-interacting module, while the CCT domain is responsible for nuclear localization and DNA binding [9,10]. These structurally conserved domains, the B-box and CCT, harbor crucial amino acid residues essential for the functionality of *COL* genes, and mutations within the B-box domain can potentially disrupt *COL* gene function [11]. In *Arabidopsis*, 17 *COL*s were divided into three subgroups based on different domains [8,12]. Group I has *CO* and *COL1*–*COL5*, characterized by two B-box domains and one CCT domain. Group II includes *COL6*–*COL8* and *COL16*, featuring a single B-box domain and one CCT domain. Group III consists of *COL9*–*COL15* and contains a zinc-finger domain in addition to the B-box domain and the CCT domain [8,13].

Up to now, researches on the *COL* gene family have shown that it has many members in both dicots and monocots, for example, 17 in *Arabidopsis* [11], 16 in rice [13], 33 in *Brassica napus* [14], 22 in sunflower [15], 11 in *Medicago* [16], 26 in soybean [17], 42 in cotton [18], and 25 in banana [19]. Functional investigations have demonstrated the significant involvement of *COL* genes in regulating plant flowering time, various developmental stages, and stress response. In *Arabidopsis*, *AtCOL3* is a positive regulator of photomorphogenesis and stimulates lateral root growth, shoot meristemization, and anthocyanin accumulation [20]. *Arabidopsis* with *AtCOL5* overexpression results in earlier flowering under short-day (SD) conditions [12]. Overexpression of *AtCOL8* and *AtCOL9* delays flowering in *Arabidopsis* [21,22]. In rice, the *AtCO* homologous gene *OsHd1* (*Heading date 1*) induces flowering under short-day (SD) conditions and inhibits flowering under long-day (LD) conditions [23,24]. Overexpression of *OsCOL9* shortened flowering time by repressing the *Ehd1* pathway [25]. According to reports on stress responses, *AtCOL4* enhances tolerance to abiotic stresses such as acid, salt, and osmotic stress through abscisic acid (ABA) signaling pathways [26]. *Ghd2* (*COL* gene) mediates drought tolerance in rice by regulating senescence [27]. Soybean (*Glycine max*) *GmCOL1a* enhances salt and drought resistance by promoting the accumulation of GmP5CS protein in transgenic soybean hairy roots [28]. In addition, the transcriptional activator *MaCOL1* in bananas is implicated in fruit ripening and stress response [29].

*Andrographis paniculata* is an annual herb in the family of Acanthaceae, which has the effects of clearing away heat and detoxification, anti-inflammation and swelling, and is widely used in the treatment of many kinds of infectious diseases and venomous snake bites [30,31]. Modern pharmacological research shows that *A. paniculata* also has a variety of medicinal functions, such as anti-cancer, anti-virus, anti-tumor, antioxidant, hypoglycemic, and hepatoprotective, so it is known as the “natural antibiotic” and has become one of the most important herbs in Asia [32,33,34]. The main active ingredients of *A. paniculata* are diterpene lactones, including andrographolide (AD), neoandrographolide (NAD), 14-deoxyandrographolide (14DAP), and dehydroandrographolide (DDAD), which vary greatly in different organs, with the largest content in leaves [35]. Among them, the pharmacological effects of andrographolide are the most extensive, with its specific efficacy against bacterial upper respiratory tract infections, and it has been demonstrated to have a significant effect against COVID-19 [36]. It has been reported that the best time to harvest *A. paniculata* is at the bud stage, when its content of andrographolide reaches its highest; so, the flowering time of *A. paniculata* has a great influence on the yield [37].

With the continuous expansion of the clinical application of *A. paniculata*, there is a need to study high-quality and high-yield *A. paniculata*. *A. paniculata* has been documented to exhibit high sensitivity to salt stress [38], which affects the synthesis of the key secondary metabolite of *A. paniculata*, diterpene lactones, thereby affecting its growth and yield [39]. Furthermore, the modulation of plant growth regulators and hormones can influence plant development and physiological processes, consequently affecting crop yield, quality, and stress resilience through comparable biological and physiological mechanisms [40,41]. As stimulants, phytohormones are involved in signaling networks that regulate the synthesis of secondary metabolites. It has been reported that phytohormones can increase the level of andrographolide in *A. paniculata* by upregulating the expression of genes related to terpene metabolism [42,43,44,45]. Hence, understanding the plant’s stress response mechanisms and identifying genes involved in hormone regulation can facilitate the investigation and selection of stress-tolerant, high-yielding variants of *A. paniculata*.

The current complete assembly of the *A. panicuulata* genome provides a resource for studying the molecular mechanisms and key genes involved in *A. paniculata* biosynthesis [37,46]. Although the *COL* gene family is well-studied in many species, focusing mainly on light regulation and circadian rhythms in plants, its important role in growth and development and stress responses has not been extensively studied, and there is currently no systematic analysis and study of COL transcription factors in *A. panicuulata*. Therefore, in this study, we identified nine *COL*s by bioinformatics methods based on the whole genome sequence information of *A. paniculata* and analyzed in depth their genetic structures, cis-regulatory elements within promoters, evolutionary relationships, and expression profiles. In addition, combining transcriptomic analysis, yeast heterologous expression, and qRT-PCR techniques, we investigated the *ApCOL* gene family, revealing its pivotal roles in responding to abiotic stress (specifically salt stress) and hormone regulation. These findings provide novel insights and candidate genes for the enhancement of *A. paniculata* through breeding programs.

## 2. Results

### 2.1. Distribution, Characterization, and Expansion of the Nine Identified ApCOL Genes

We identified nine *COL* genes in the *A. paniculata* genome and predicted and analyzed their physical location and physicochemical properties. Detailed information is listed in Table 1, including gene name, gene ID, chromosomal location, gene length, protein length, molecular weight (MW), isoelectric point (pI), and subcellular localization prediction. In addition, the classification, location, exon number, and BBOX and CCT structural domains of *ApCOL* members were added to Table S1. *ApCOL*s were renamed from *ApCOL01* to *ApCOL09*, depending on the location of the gene on the chromosome. The gene length of *ApCOL*s ranged from 1212 bp (*ApCOL07*) to 5067 bp (*ApCOL03*), while the length of its corresponding protein ranges from 329 aa (ApCOL03) to 433 aa (ApCOL08). The online program predicted the MWs of COL proteins in the range of 35.32 kDa (ApCOL03) to 48.41 kDa (ApCOL08), with theoretical pIs ranging from 5.1 (ApCOL02 and ApCOL05) to 6.81 (ApCOL07). In addition, the subcellular localization predicted that the majority of COL proteins were located in the nucleus (77.7%), with the remainder (ApCOL02 and ApCOL07) being located in the chloroplast (Table 1).

**Figure 1:**
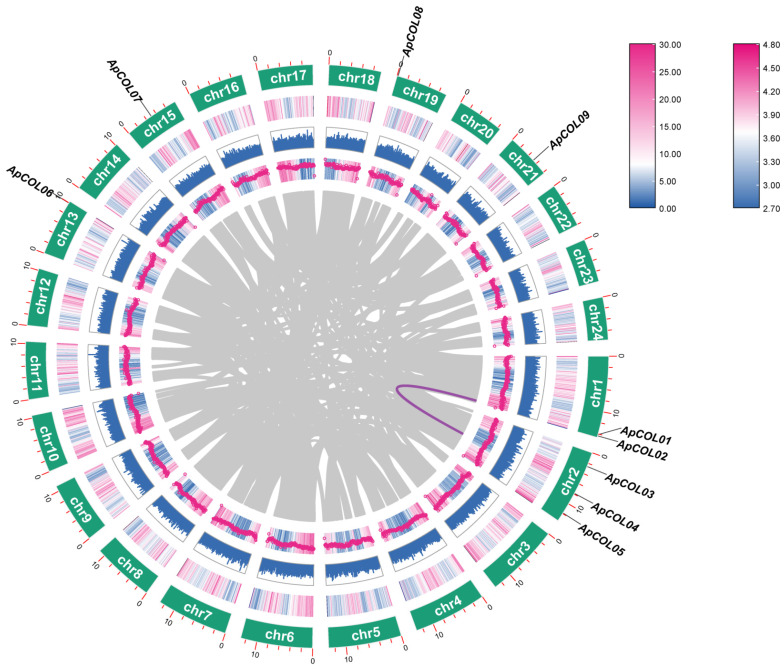
Interchromosomal homology analysis of *ApCOL* gene in the *A. paniculata* genome. The purple line represents the replication events of the *ApCOL* genes, and the gray line represents the syntenic blocks in the *A. paniculata* genome.

In addition, nine *ApCOL* genes were localized on six chromosomes and were unevenly distributed. They were mainly concentrated on Chr1(2) and Chr2(3), and the remaining *ApCOL*s were mono-distributed on Chr13, -15, -19, and -21, respectively. Due to the low number of identified *ApCOL* genes, we analyzed the *COL* gene duplication events in the *A. paniculata* genome to understand the amplification mechanism of the *ApCOL* gene family. The results showed that only one pair of duplicated genes (*ApCOL01*/*ApCOL04*) was identified, and it might have been generated by segmental duplication or whole genome duplication (WGD) (Figure 1).

### 2.2. Phylogenetic Analysis and Well-Defined Classification of Andrographis paniculata COL Genes

**Figure 2:**
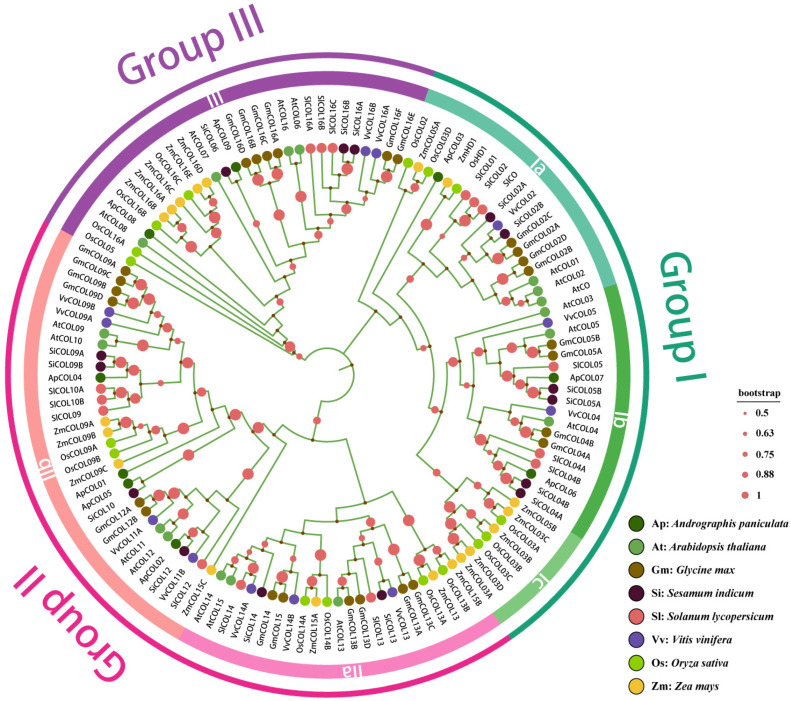
NJ Phylogenetic tree of eight plant COL proteins. The phylogenetic tree is divided into three groups and six subgroups, each marked with different colors. Circular nodes represent Bootstrap values above 50%.

**Figure 3:**
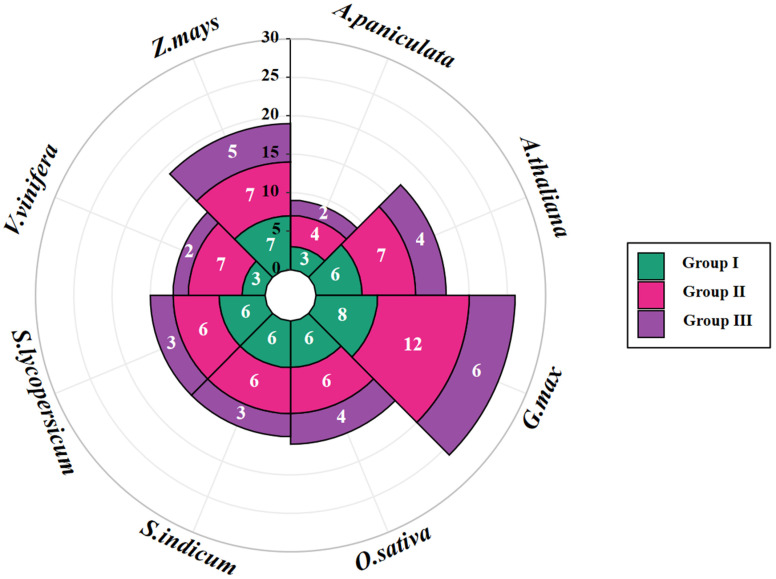
The Nightingale Rose Chart calculates the number of *COL* members in eight species and shows the number of members in the three groups. The eight plants include *A. paniculata*, Arabidopsis (*A.thaliana*), soybean (*G. max*), sesame (*S. indicum*), tomato (*S. lycopersicum*), grape (*V. vinifera*), rice (*O. sativa*), and maize (*Z. mays*).

To understand the evolutionary relationships of *COL* gene families among different species, we constructed a multi-species phylogenetic tree by the neighbor-joining method, which included both dicotyledons (*A. paniculata*, 9; *Arabidopsis*, 17; soybean, 26; sesame, 15; tomato, 15; grape, 12) and monocotyledons (rice, 16; maize, 19). Phylogenetic analyses showed that during evolution, *COL* genes strongly clustered into three clusters: groups I, II, and III (Figure 2). This is consistent with previous reports [13]. According to the characteristics of each clade, groups I (Ia, Ib, and Ic) and II (IIa and IIb) were further subdivided into subgroups, while group III remained unaltered. The distribution of ApCOL proteins across the three groups was uneven, with group III exhibiting the fewest *ApCOL* members. Each subgroup, with the exception of subgroups Ic and IIa, contained at least one *ApCOL* member. Subsequently, we counted the number of *COL* members for the eight species in each group and found that the group III also had the lowest number of COL proteins among the eight plants (Figure 3).

From the phylogenetic tree, it can be seen that the *COL* genes of the two monocotyledonous plants (rice and maize) were tightly clustered, particularly the Ib and Ic subgroups, which exclusively harbor *COL* family genes from dicotyledonous and monocotyledonous plants, respectively. This suggests independent evolution of *COL* genes in dicotyledonous plants and raises the possibility of independent amplification of the Ib and Ic subgroups post-divergence. Moreover, *ApCOL* genes consistently showed close clustering with *COL* genes of *Sesamum indicum*, which may be due to the fact that both have a recent common ancestor or have experienced similar selective pressures and possess a large number of homologous genes, thus showing significant similarity (Figure 2).

### 2.3. Gene Structure and Conserved Motifs Analysis of ApCOLs

**Figure 4:**
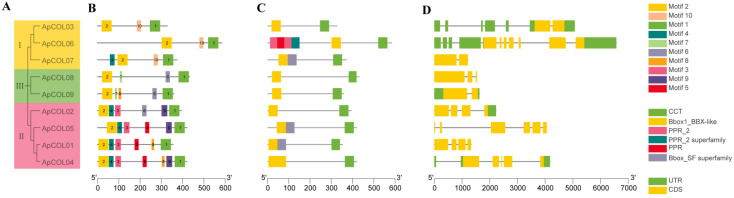
Phylogenetic tree, conserved motifs, and gene structure analyses of *A. paniculata ApCOL* genes. (**A**) Evolutionary relationships of the nine *ApCOL* genes. (**B**) Conserved motif composition of ApCOL proteins. (**C**) Distribution of conserved protein structural domains of *ApCOL*s. (**D**) Exon–intron structure analysis of *ApCOL*s. Exons and introns are represented by yellow boxes and gray lines, respectively.

To validate the phylogenetic analysis grouping results, we utilized the MEME program to predict the structural characteristics of ApCOL protein sequences, identifying 10 conserved motifs ranging in length from 11 to 49 amino acids (Figure 1 and Figure 4B, Figure S1). All *COL* members of *A. paniculata* contain two conserved structural domains: the N-terminal B-box structural domain (motif 2) and the CCT structural domain near the C-terminus (motif 1). In addition to these two representative conserved structural domains, motifs from the same group tend to be highly conserved. For instance, all members of group I contain motifs 1, 2, and 10, with motif 10 exclusive to this group. Group III members share four motifs, with motif 7 being specific to group II. Most group II members feature six motifs, with motifs 1, 2, 3, and 4 shared by four members, and motifs 3, 5, and 9 unique to this group. However, we also found some differences within the same group. The closest *ApCOL03* and *ApCOL06* in group I contain consistent motif distributions and lengths, whereas *ApCOL07* and *ApCOL09* in group III contain motifs that are different from the other members of the same group. It is noteworthy that all four members of group III are slightly different.

According to the results of multiple sequence alignments, all ApCOL proteins were found to possess at least one B-box and CCT structural domain (Figure 4C). In group I, only *ApCOL07* contained two B-box structural domains and one CCT structural domain. Group II members uniformly displayed two B-boxes and one CCT domain, with the exception of *ApCOL02*, which featured one of each domain. Group III members were characterized by two B-boxes and one CCT structural domain. These results are broadly in line with previous findings that *COL* genes are classified into three groups based on the number and type of B-box structural domains [13], but there are some differences in group membership, which may be due to complex evolutionary mechanisms that result in the gain or loss of structural domains that promote functional specialization. Notably, *ApCOL06* contained four PPR structural domains, known to be a prominent protein family in flowering plants, with significant roles in plant growth and development. The biological functions of PPR proteins within *A. paniculata COL* members warrant further investigation [47].

We also analyzed the intron–exon structures of *ApCOL*s and showed a high degree of variation among the nine members (Figure 4D). The majority of genes exhibited between one to six introns and two to six exons, with *ApCOL06* displaying the highest count of ten introns and seven exons. Nonetheless, the length and quantity of exons and introns were observed to be more similar within the same subgroup compared to across different subgroups.

### 2.4. Cis-Regulatory Elements and Functional Analysis of the ApCOL Promoter Regions

**Figure 5:**
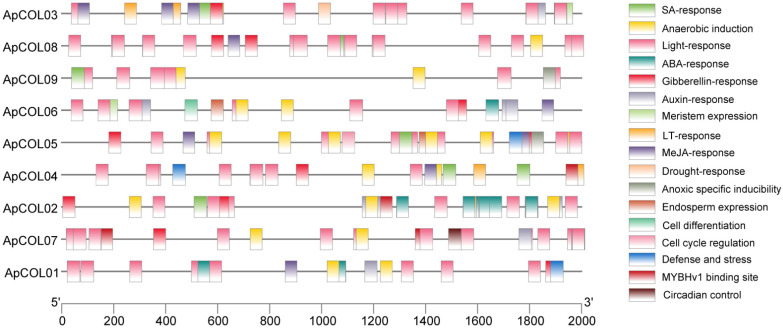
Distribution of cis-regulatory elements in the 2000 bp sequence of the promoter region of the *ApCOL* gene. Each cis-regulatory element is indicated by a different color and is positioned in the same position as the corresponding position of the promoter.

In order to explore the potential functions of the *ApCOL* gene and its expression regulation under abiotic stresses, the cis-regulatory elements (CREs) within the 2000 bp promoter sequence upstream of *ApCOL* were identified, and 17 elements associated with specific functions were selected for analysis (Figure 5). These CREs were broadly categorized into four groups: light responses, abiotic stress responses, hormone responses, and growth regulation. This is consistent with predictions in other species, all of which contain predominantly these four CREs [14,15]. The promoters of all *ApCOL* genes were found to be enriched in light-responsive elements, with a range from 6 (*ApCOL06*) to 18 (*ApCOL02*/*08*), indicating a potential role of *ApCOL* genes in regulating light responses in flowering plants (Table S3. The phytohormone response elements identified included auxin (IAA), gibberellin (GA), salicylic acid (SA), abscisic acid (ABA), and methyl jasmonate (MeJA). Among the eight *ApCOL*s analyzed, all exhibited a minimum of three hormone response elements, with the exception of *ApCOL09,* which possessed only one element related to salicylic acid response. In addition, multiple CREs involved in abiotic stress responses were detected. Notably, *ApCOL*s (*ApCOL02*, -*04*, and -*07*) were found to interact with MYBHv1, suggesting their involvement in drought response modulation. (Table S3). Growth regulatory elements, such as meristematic tissue expression, cell cycle regulation, and endosperm expression, were relatively few, particularly in relation to *ApCOL07*. Circadian control elements detected in *ApCOL07* corresponded to the expression pattern of the *COL* gene.

Combined with the results of the above analyses, the *ApCOL* genes were subjected to Gene Ontology (GO) enrichment analysis to further understand their functions and regulatory mechanisms. The biological functions were classified into three categories: biological processes (BPs), molecular functions (MFs), and cellular components (CCs) (Table S4, Figure S2). Biological processes (BPs) included DNA binding and ions and organic cyclic compounds binding, as well as transcriptional regulatory activity photoperiod regulation, long-day regulation, flowering regulation, circadian rhythms, and responses to various stimuli, including light and abiotic stress. Molecular function (MF) included DNA binding and ions and organic cyclic compounds binding, as well as transcriptional regulatory activity. Cellular component (CC) annotations indicated that all gene products were localized in the nucleus and organelles, which is in full agreement with our earlier prediction of subcellular localization. These findings are in agreement with the cis-regulatory element analysis conducted.

### 2.5. Interspecies Syntenic Analysis

**Figure 6:**
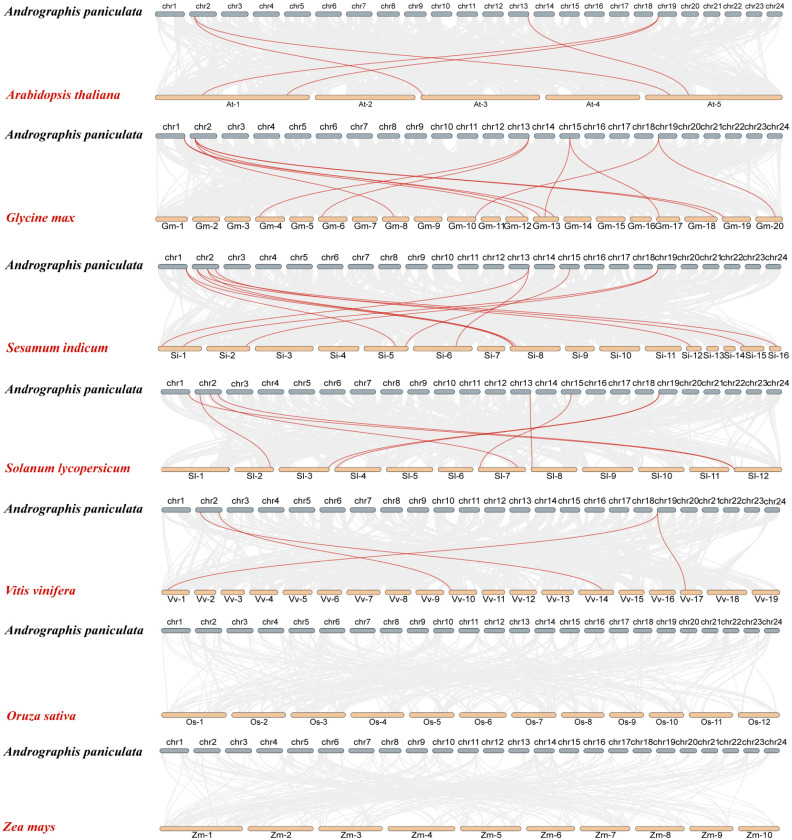
Synteny relationship analysis of *COL* genes between *A. paniculata* and other plants. Gray lines in the background represent collinear blocks in the *A. paniculata* and other plant genomes, and green lines indicate synthetic *COL* gene pairs.

Interspecies syntenic analysis enhances the study of gene family evolutionary relationships. Therefore, we analyzed the synteny of *A. paniculata* with seven other plant species, including five dicotyledons (*Arabidopsis*, soybean, sesame, tomato, and grape) and two monocotyledons (rice and maize) (Figure 6). The results showed that *ApCOL*s had 5, 12, 12, 8, and 4 pairs of homologous genes with *Arabidopsis*, soybean, sesame, tomato, and grape, respectively (Table S5). Among them, the number of homologous genes between *A. paniculata* and *S. indicum* was the relative highest, which is consistent with our previous speculation. However, there were no homologous gene pairs between *ApCOL*s and rice and maize. These results suggest that the *ApCOL* gene family likely emerged post the dicotyledon-monocotyledon divergence, showcasing close ties to dicotyledon evolution. Syntenic analysis of *A. paniculata* alongside five dicotyledons (excluding *Arabidopsis*) disclosed that *COL* genes in groups I, II, and III participated in collinear gene pair formations. Particularly, the collinearity of *ApCOL08* in group III was in the form of a pair of multiple pairs, implying a high conservation level across diverse dicotyledonous plant species and potentially pivotal evolutionary roles. In particular, *A. paniculata* displayed a one-to-many expression pattern of homologous genes with soybean, notably exemplified by *ApCOL03*, which exhibited homology with four soybean *COL* genes and covaried with the other four species, emphasizing its significance in *COL* gene family evolution (Table S5). These results have important implications for predicting gene function.

### 2.6. Expression Profiles of ApCOL Genes in Different Tissues

**Figure 7:**
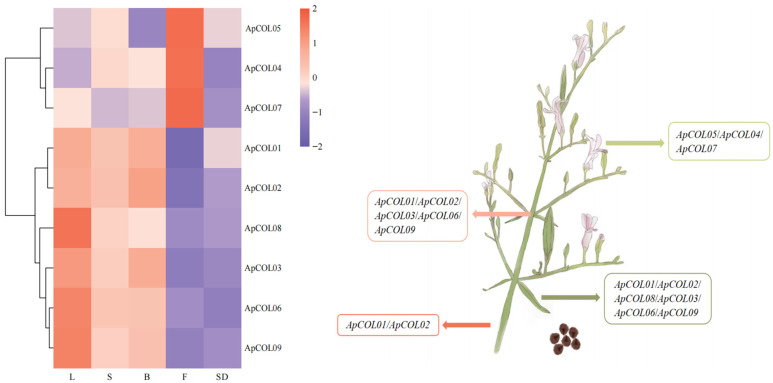
Tissue-specific expression profiles of *ApCOL* genes in different tissues of *A. paniculata*. The logarithm of FPKM values was normalized for creating heatmaps. Different colors represent gene expression levels, with high expression levels approaching red and low expression levels approaching green. Genes with relative expression values > 0.3 in each tissue are listed in the four squares of the right-hand panel, and no genes were expressed in seeds. L: Leaves; S: Stem; B: Branch; F: Flowers; SD: Seed.

To explore the potential role of *ApCOL* genes in the growth and development of *A. paniculata*, RNA-seq data from five distinct tissues, including leaves, stems, flowers, branches, and seeds, were sequenced using the Illumina high-throughput sequencing platform. Analysis revealed differential expression patterns of *ApCOL*s across various tissues, while hierarchical clustering analysis indicated similar expression profiles within the same cluster (Figure 7). Firstly, *ApCOL04*, *ApCOL05*, and *ApCOL07* were significantly expressed only in flowers but not in other tissues, with certain specific expression characteristics. Conversely, *ApCOL01* and *ApCOL02* were expressed in leaves, stems, and branches, but not in flowers and seeds. Moreover, *ApCOL03*, *ApCOL06*, and *ApCOL09* were highly expressed in leaves, barely expressed in stems, less expressed in branches, and not expressed in flowers and seeds. In particular, *ApCOL08* demonstrated strong expression in leaves and minimal expression in other tissues, while all nine *ApCOL*s were found to have negligible expression in seeds. These findings suggest the involvement of *ApCOL*s in diverse growth and developmental processes, excluding seed development, with pivotal roles in leaf and flower development. This finding is consistent with that with other species, where most *COL* genes are highly expressed in plant leaves [14,48,49]. Additionally, *ApCOL* members sharing similar expression patterns likely possess analogous functionalities.

### 2.7. Expression Profiles of ApCOL Genes Under Hormone Treatment

**Figure 8:**
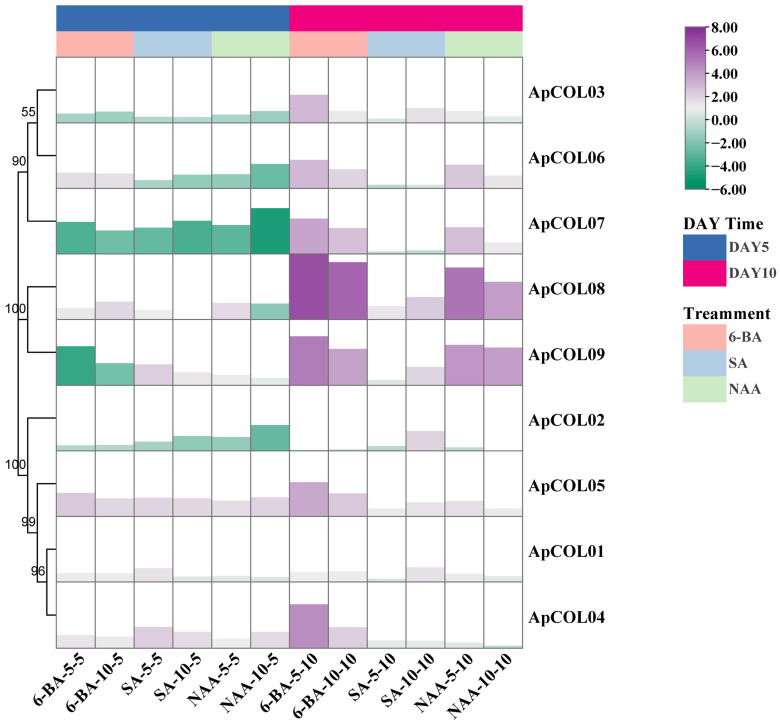
The expression profile of *ApCOL* genes in the leaves of *A. paniculata* under three PGR treatments (6-BA, SA, and NAA) was analyzed by qRT-PCR. The relative expression levels of genes were calculated by using the 2^−ΔΔCt^ method. The UBC gene serves as an internal reference gene. The results were visualized as log_2_-fold changes. 6-BA-5-5/6-BA-5-10: *A. paniculata* treated with 5 μM 6-BA for 5 and 10 days; 6-BA-10-5/6-BA-10-10: *A. paniculata* treated with 10 μM 6-BA for 5 and 10 days; SA-5-5/SA-5-10: *A. paniculata* treated with 5 μM SA for 5 and 10 days; SA-10-5/SA-10-10: *A. paniculata* treated with 10 μM SA for 5 and 10 days; NAA-5-5/NAA-5-10: *A. paniculata* treated with 5 μM NAA for 5 and 10 days; NAA-10-5/NAA-10-10: *A. paniculata* treated with 10 μM NAA for 5 and 10 days.

Subsequent to our previous analysis, it was observed that the promoter region of the *ApCOL* gene harbored a significant number of phytohormone response elements (Figure 5). Consequently, we investigated the expression profiles of *ApCOL*s under the influence of various plant growth regulators (PGRs)—6-benzylaminopurine (6-BA), salicylic acid (SA), and α-naphthaleneacetic acid (NAA)—and at different concentrations for 5 and 10 days using qRT-PCR (Figure 8, Table S7). Preliminary results indicate that changes in the three PGR concentrations had little effect on the genes, except for seven *ApCOL* genes under SA treatment on day 10, where most showed slight downregulation at high concentrations. Notably, the expression of *ApCOL08* decreased by more than threefold at high concentrations compared to low concentrations. Nevertheless, notable variations were observed in the response of *ApCOL* genes to PGRs at different treatment durations. Specifically, in both concentrations of the 6-BA solution, a substantial increase in gene expression was evident after a 10-day treatment compared to a 5-day treatment, with all but *ApCOL01* and *ApCOL02* showing significant upregulation (log_2_ ≥ 2). Among these, *ApCOL08* exhibited the highest expression levels (log_2_ ≥ 6). In addition, *ApCOL06*, -*07*, -*08,* and -*09* displayed significant upregulation following 10 days of treatment with varying concentrations of NAA. However, *ApCOL07* experienced substantial downregulation (log_2_ ≤ −2) within the first 5 days of treatment across all three PGRs. *ApCOL01*, -*04*, and -*05* demonstrated upregulation in all 12 treatment conditions, while *ApCOL02* predominantly exhibited downregulation. Overall, a prevailing trend of upregulation in gene expression changes was observed, suggesting that exogenous hormones had a discernible impact on the expression of *ApCOL*s in *A. paniculata*.

### 2.8. Expression Profiles of ApCOL Genes Under Salt Stresses

**Figure 9:**
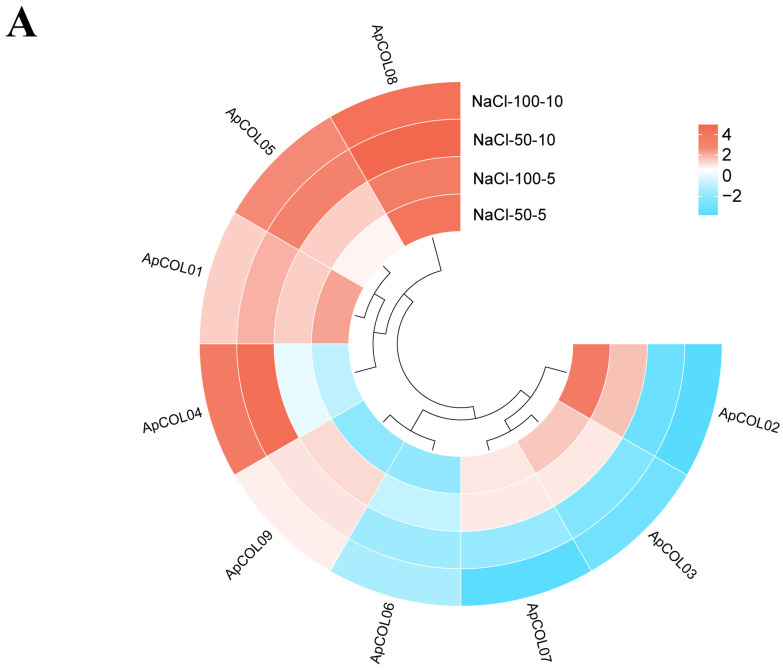

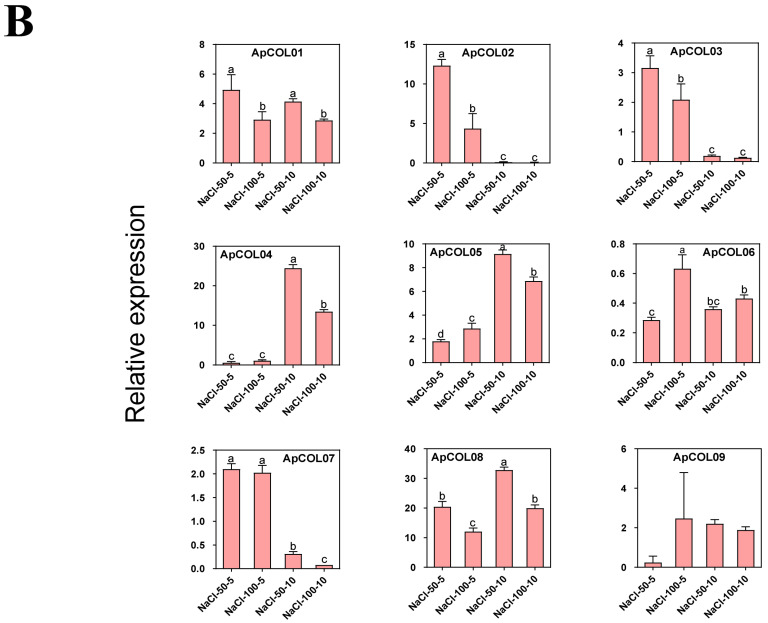
Expression analysis of *COL* genes in *A. paniculata* leaves under salt treatment. Expression analysis of *ApCOL*s in the leaves of *A. paniculata* under salt stress. (**A**) The expression profile of *ApCOL* genes under salt treatment was analyzed by qRT-PCR. The relative expression levels of genes were calculated by using the 2^−ΔΔCt^ method. The UBC gene serves as an internal reference gene. The results were visualized as log_2_-fold changes. (**B**) Relative expression of nine *ApCOL*s in response to salt stress. The histogram was presented by the mean and standard error of the data. Lowercase letters “a, b, c, d” indicate a significant level of 0.05, and different letters indicate significant differences between groups (*p* < 0.05). NaCl-50-5/NaCl-50-10: *A. paniculata* treated with 50 mM NaCl for 5 and 10 days; NaCl-100-5/NaCl-100-10: *A. paniculata* treated with 100 mM NaCl for 5 and 10 days.

To further elucidate the involvement of *ApCOL* genes in response to various stresses, we conducted an analysis of the expression profiles of *ApCOL*s under different salt concentrations and treatment times (Figure 9A, Table S6). The results showed distinct expression patterns of *ApCOL*s in leaves across diverse treatment conditions. *ApCOL01*, -*05*, and -*08* exhibited significant upregulation (log_2_ ≥ 2) under all four treatments. For instance, *ApCOL01* displayed considerable upregulation across various treatment durations and low salt concentrations, while *ApCOL05* showed significant upregulation after a 10-day treatment under varying salt concentrations, as did *ApCOL04*. Particularly noteworthy was the strong upregulation of *ApCOL08* under all treatment conditions, with pronounced induction observed under NaCl-50-10 conditions (log_2_ ≥ 5). In addition, the expression levels of *ApCOL02*, *ApCOL03*, *ApCOL07*, and *ApCOL09* were significantly downregulated (log_2_ ≤ −2) (Table S7).

To better investigate the response mechanism of *ApCOL* to salt stress, we conducted an analysis of the relative expression levels of nine *ApCOL*s that exhibited significant changes under four distinct salt treatment conditions (Figure 9B). The results showed that six genes (*ApCOL01*, -*02*, -*03*, -*04*, -*07*, and -*08*) were significantly downregulated after 5 days of salt treatment, while *ApCOL06* showed significant upregulation. Moreover, *ApCOL05* exhibited upregulation at 10 days of salt treatment, followed by downregulation at higher salt concentrations. The expression patterns of three genes, *ApCOL04*, -*05*, and -*08*, were significantly upregulated, while *ApCOL02*, -*03*, and -*07* were downregulated at varying treatment durations. *ApCOL06* demonstrated a significant decrease in expression over time at high salt concentrations, with no significant difference observed at lower concentrations. There were no significant differences in the expression levels of *ApCOL01* and *ApCOL09*. In addition, *ApCOL08* displayed a substantial upregulation (more than 30-fold) in leaves subjected to high salt concentrations for 10 days.

### 2.9. Subcellular Localization of Key ApCOL Proteins

**Figure 10:**
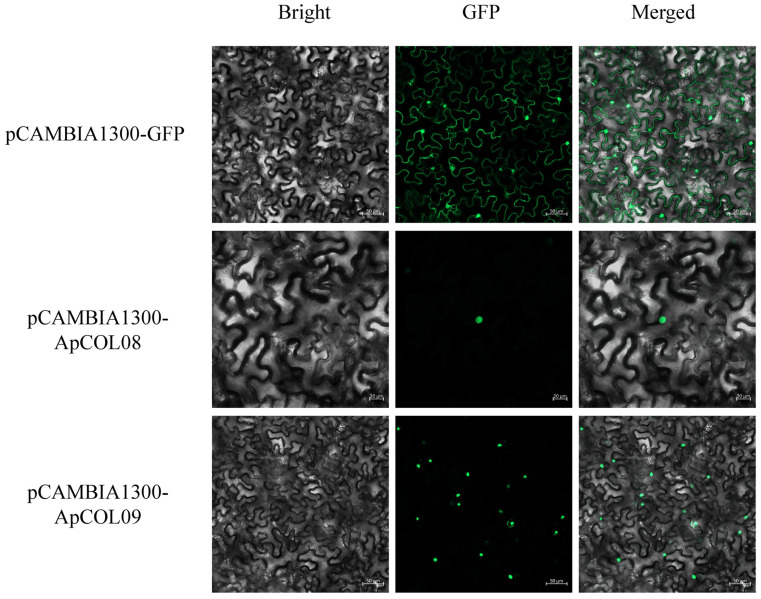
Subcellular localization of the ApCOL08 and ApCOL09 proteins in *A. paniculata*.

To determine the subcellular localization of ApCOL proteins to explore their function further, we fused *ApCOL08*/*09* with a green fluorescent protein (eGFP). The results showed that the green fluorescence signals of both GFP-ApCOL08 and GFP-ApCOL09 were localized only to the nucleus, in contrast to the green fluorescence of the pCAMBIA1300-GFP empty vector, which was present in all parts of the cell (Figure 10). The results were consistent with previous BioSignal predictions.

### 2.10. Heterologous Expression Verification of ApCOL08 Salt Tolerance Function in Yeast

**Figure 11:**
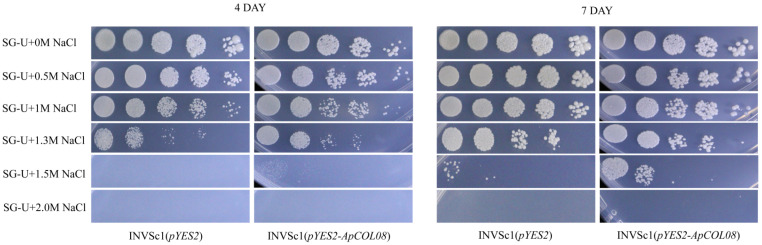
Comparison of growth between INVSc1 (pYES2-*ApCOL08*) and INVSc1 (pYES2) under treatment with six concentrations of NaCl (0 M, 0.5 M, 1 M, 1.3 M, 1.5 M, 2 M). Photos were taken after incubating at 30 °C for 4 d and 7 d, respectively.

The plasmid pYES2 is designed to induce the expression of recombinant proteins in yeast and features the yeast GAL1 promoter. The plasmid pYES2 is specifically engineered for inducing the expression of recombinant proteins in yeast and features the yeast GAL1 promoter. This promoter enables the expression of proteins of interest at high levels by utilizing galactose-inducible proteins in *Saccharomyces cerevisiae*. Additionally, pYES2 includes the CYC1 terminator, which is glucose-suppressed and serves to effectively terminate mRNA transcription [50,51]. Given that *ApCOL08* shows significant induction in response to various salt stresses, we selected its heterologous expression in yeast as a preliminary study of its biological function (Figure 11).

A comparative analysis of the growth patterns of recombinant pYES2-*ApCOL08* and pYES2 empty vector yeast (INVSc1) strains under varying salt stress concentrations revealed intriguing findings. On day four, it was observed that INVSc1 (pYES2) exhibited better growth than INVSc1 (pYES2-*ApCOL08*) under 0 M, 0.5 M, and 1 M NaCl stress conditions. Under 1.5 M NaCl stress, a slight growth was observed in INVSc1 (pYES2-*ApCOL08*), while INVSc1 (pYES2) did not grow. Both yeast strains demonstrated no growth under 2 M NaCl stress. On the seventh day, a more pronounced growth trend was observed between the two yeast strains, and the contrast between the two yeasts was even more pronounced, especially under 1.3 M and 1.5 M NaCl stress, where the growth of INVSc1 (pYES2-*ApCOL08*) was significantly higher than the growth of INVSc1 (pYES2) under 1.5 M NaCl treatment (Figure 11). These results indicated that heterologous expression of the *ApCOL08* gene in yeast improved tolerance to NaCl stress and further confirmed that *ApCOL08* plays an important role in salt stress tolerance.

## 3. Discussion

The *COL* gene family plays a crucial regulatory role in the photoperiodic response and flowering in plants. Numerous studies have reported the presence of *COL* genes in various plant species, indicating their involvement in regulating diverse developmental processes, abiotic stress responses, and homeostatic mechanisms in plants [26,52,53,54]. This includes key regulation of flowering time control, circadian rhythms, photosynthesis, shade avoidance responses, and formation of light and dark morphology during development [55]. In this study, we identified nine *ApCOL* genes from *A. paniculata* and classified them into three subgroups based on the *Arabidopsis* grouping (Table S1). *A. paniculata* contains fewer *COL* genes compared with other plant species, such as the seven plants in the previous syntenic analysis, *Arabidopsis*, soybean, sesame, tomato, grape, rice, and maize, which contain 17, 26, 15, 15, 12, 16, and 19 *COL* genes, respectively [56,57,58,59].

In addition, the genome sizes of these plant species varied considerably; however, it is noteworthy that there was no significant correlation observed between the number of *COL* genes and genome size. For instance, *Arabidopsis* has a genome size of 119 Mb, while the genome size of tomato is nearly seven times larger than that of *Arabidopsis*. Despite this difference in genome size, the number of *COL* genes in tomato is not significantly different from that in *Arabidopsis* (Table S2). Additionally, we noted that the genome sizes and *COL* gene numbers of sesame and *A. paniculata* are relatively similar, and they are closely related in evolution. This suggests that the number of *COL* gene families is more closely associated with species evolution rather than genome size [60]. Based on these findings, it is hypothesized that the *ApCOL* genes of *A. paniculata* have been relatively conserved during evolution, or, alternatively, some members of the *COL* gene family may be absent from the *A. paniculata* genome.

Subsequent investigations into *COL* genes have revealed that genome duplication events are associated with the expansion of gene family members. In this study, a pair of fragment duplication genes (*ApCOL01*/*ApCOL04*) was identified in the *A. paniculata* genome (Figure 1). Intriguingly, this duplicated gene pair exhibits homology with corresponding genes in sesame and tomato, indicating a close evolutionary relationship with *A. paniculata* (Table S5). These findings suggest that segmental duplication may play a significant role in the evolution of *A. paniculata COL* genes, and that these genes and their functions are relatively conserved among closely related species.

Conserved protein motifs and gene structures serve as bases for predicting evolutionary relationships among species and gene functions [61,62]. Members belonging to the same subfamily typically exhibit similar gene structures and motif distributions, suggesting comparable functions [60]. Within the *COL* family, proteins commonly possess two conserved structural domains: the B-box structural domain and the CCT structural domain [13]. All ApCOL proteins contain these two structural domains, with both group I and group II proteins featuring two B-boxes: Bbox1 and Bbox2. The exception is *ApCOL02*, which is speculated to have lost Bbox2 during the evolutionary process of *A. paniculata*, or where Bbox2 in these genes may have arisen from a duplication event of Bbox1 [63] (Figure 4C).

The proteins of group III are characterized by the presence of only one B-box and one CCT, which is consistent with the phylogenetic class II of COL proteins, and based on previous speculations, group III may be an early conserved protein. Analysis of gene structure reveals significant variation in sequence length among *ApCOL* genes. Studies have demonstrated that the number of introns can impact gene expression levels, with fewer introns associated with higher gene expression levels. For instance, *ApCOL07*, -*08*, and -*09* exhibit relatively short protein sequences and fewer introns, potentially indicating rapid induction under stress conditions [48,64,65,66,67] (Figure 4D and Figure 9B, salt stress). Conversely, *ApCOL06*, which has the highest number of introns, exhibits the lowest expression level (Figure 9A). The diverse gene structures of *ApCOL* family members offer valuable insights for investigating the evolution and protein function of *ApCOL*s.

*COL* genes have been reported to exhibit wide expression across various plant tissues, with significant tissue specificity [68]. In our study, we observed the predominant expression of most *ApCOL*s in leaves and branches, particularly high expression levels of *ApCOL03*, -*06*, and -*09* in leaves, and exclusive leaf expression of *ApCOL08* at a high level (Figure 7). Leaves are known as primary organs for sensing photoperiodic signals, and the high expression of *ApCOL* in leaves suggests its role in activating *FT* transcription to promote flowering [69,70]. Additionally, apart from *ApCOL08*, we identified three genes (*ApCOL04*, -*05,* and -*07*) with pronounced transcript levels specific to flowers, indicating their potential significance in flower growth and development. Notably, the absence of *ApCOL* expression in seeds implies a lack of involvement in *A. paniculata* seed development (Figure 7).

Cis-regulatory elements in the promoter region play a crucial role in the regulation of gene expression under the influence of various factors [71,72]. Numerous light-responsive elements have been identified in the promoter region of the *ApCOL* gene, and many reports have shown that the *COL* gene is a key regulator in photoperiod-mediated regulation of flowering, suggesting that the *A. paniculata COL* gene is sensitive to photoperiodic changes [49,69,73]. Furthermore, the identification of response elements to various exogenous phytohormones and abiotic stresses in the promoter region implies a potential inducibility of *ApCOL* gene expression by these stimuli (Figure 5).

To date, *COL* has been extensively investigated in the context of light regulation and circadian rhythms in plants, but its responses to exogenous hormones and abiotic stresses remain relatively understudied. In *Arabidopsis*, it has been observed that ABA, salt stress, and osmotic stress can upregulate the expression of *AtCOL4*, thereby improving salt tolerance [26]. In rice, the *Ghd7* gene, a homolog of *CO-like* genes, not only controls plant height, heading stage, and grain yield, but also plays a role in enhancing stress tolerance [27]. Additionally, *OsCOL9* has been found to interact with OsRACK1 via salicylic acid and ethylene signaling pathways, contributing to elevated resistance in rice [52].

In our study, we employed qRT-PCR to anticipate the expression patterns of *ApCOL*s in specific environmental conditions. Our findings revealed that alterations in hormone concentrations did not impact the expression levels of *ApCOL*s, however, their expression was notably upregulated over the course of treatment (Figure 5). *ApCOL08* and *ApCOL09* exhibited similar expression profiles, especially in response to 6-BA and NAA treatments, displaying significant induction. *ApCOL01*, -*04*, and -*05* demonstrated varying degrees of upregulation across all treatment conditions, indicating potential involvement in the whole process of hormone signaling crosstalk. The expression of *ApCOL03*, -*06,* and -*07* was initially suppressed following treatment initiation. Notably, the majority of *ApCOL*s exhibited robust induction following prolonged exposure to 6-BA. These observations suggest that *ApCOL*s may serve as regulatory elements involved in phytohormone modulation through synergistic mechanisms.

Several abiotic factors, including soil salinity, have been identified as serious impediments to the productivity of medicinal plants. Salt stress stands out as a prominent abiotic factor contributing to diminished plant growth and productivity [74,75]. In the present investigation, the expression patterns of the majority of *ApCOL*s were influenced by two different concentrations and durations of salt treatments (Figure 9A). Of *ApCOL*s, 67% exhibited elevated expression levels under low salt concentrations compared to high salt concentrations, while 56% of *ApCOL*s showed higher expression levels under short-term salt stress rather than long-term salt stress conditions (Table S7). Noteworthy is the expression behavior of *ApCOL08* under salt stress, showcasing significant responsiveness to varying concentrations and treatment durations, suggesting its potential as a key player in salt stress tolerance within *A. paniculata* (Figure 9B). We chose *ApCOL08* and *ApCOL09* for subcellular localization. *ApCOL08* and *ApCOL09* are located in the same subgroup in the phylogeny, and not only the gene structures are similar, but the expression patterns of both are similar, so we speculate that *ApCOL08* and *ApCOL09* may have similar functions. The subcellular localization results showed localization to the nucleus, which was consistent with the prediction of BioSignal, suggesting that *ApCOL08* and *ApCOL09* proteins function in the nucleus (Figure 10).

So far, some progress has been made in the research on the genetic engineering of *A. paniculata*. Still, the complexity of the *A. paniculata* genome and the fact that the genetic transformation system of *A. paniculata* has not yet been established have created a great limitation for us in studying its gene function. Compared with heterologous expression in *Arabidopsis*, we found an efficient and more rapid means to verify plant resistance-related gene functions. In recent years, yeast heterologous expression has been used more frequently for the study of stress tolerance genes, such as in *Populus trichocarpa*, where the resistance of *PtrRZFP1*/*4*/*7* to osmotic and salt stresses was verified by observing the growth condition of *S. cerevisiae* [76]. Overexpression of *GbSOS1* enhanced the salt tolerance of yeast, thus providing evidence to support the screening of candidate genes for the cultivation of salt-tolerant *Gossypium barbadense* [77]. *TrSAMDC1* was expressed in yeast for the *Arabidopsis* in salt and cold tolerance [78]. *S. cerevisiae* has a eukaryotic expression system close to that of plants and also has the processes of glycosylation, disulfide bond formation and protein folding, and post-translational processing, which leads to the normal functioning of proteins encoded by the screened plant genes, and the probability of false positives of the validated genes for adversity resistance is greatly reduced [79]. Therefore, yeast became an ideal model for verifying gene resistance in this study. We used the INVSc1-pYES2 recombinant protein inducible expression system to heterologously express *ApCOL08* in yeast for further validation (Figure 11). Encouragingly, transformed INVSc1 yeast (pYES2) with *ApCOL08* showed enhanced tolerance to high salt stress compared with untransformed INVSc1 yeast (pYES2), confirming the qRT-PCR expression analysis and emphasizing the possible involvement of the *ApCOL08* gene in the salt stress response mechanism. Our study preliminarily verified the potential function of *ApCOL08* in *A. paniculata* salt stress, and later we will further investigate the stable transgenic *A. paniculata* method to fully elucidate the nuclear regulatory mechanism of its gene function.

## 4. Materials and Methods

### 4.1. Identification and Update of the COL Genes

The relevant genome files of *A. paniculata* were downloaded from the National Center for Biotechnology Information database (NCBI, PRJNA421867) [46]. The HMM models for CCT (PF06203) and B-box (PF00643) were obtained from the Pfam database (http://pfam.xfam.org/ (accessed on 12 February 2024)) [80] and compared in the *A. paniculata* genome by HMMER 3.3.2 (http://hmmer.org/ (accessed on 12 February 2024)), with an E-value of 10^−3^, thus screening for potential ApCOL protein sequences [81]. Redundant sequences were removed, and the results were used to create specific HMM models of ApCOL proteins for secondary searches. Meanwhile, the AtCOL protein sequences (https://www.arabidopsis.org/ (accessed on 20 February 2024)) were served as query sequences for BLAST analysis of ApCOL, with an E-value of 10^−10^. The CDD database (https://www.ncbi.nlm.nih.gov/cdd/ (accessed on 20 February 2024)) was applied for multiple sequence analysis [82], and only the genes containing both of these domains were eventually identified as the candidate genes. In addition, the SMART database (http://smart.embl.de/ (accessed on 22 February 2024)) was applied to verify the integrity of the ApCOL protein [83]. The sequences of the remaining species, i.e., *Arabidopsis* (*Arabidopsis thaliana*), soybean (*Glycine max*), sesame (*Sesamum indicum*), tomato (*Solanum lycopersicum*), grape (*Vitis vinifera*), rice (*Oryza sativa*), and maize (*Zea mays*), were obtained from the Ensembl Plants with the above method of retrieval and update (http://plants.ensembl.org/index.html (accessed on 1 March 2024)).

### 4.2. Chromosomal Location and Syntenic Analysis of ApCOLs

The *COL* genes were renamed based on their chromosomal location, and the Perl script was employed for the collection of distribution information. Syntenic analysis was performed via the Multiple Collinearity Scan toolkit (MCScanx https://github.com/wyp1125/MCScanX (accessed on 20 February 2024)) for determining the duplication patterns of *COL* genes in the *A. paniculata* genome with default parameters (E-value set to 10^−10^) [84]. Inter-species syntenic analyses were conducted to anticipate the evolutionary preferences of *COL* family genes (Dicotyledonous: *Arabidopsis*, soybean, sesame, tomato, and grape; Monocotyledonous: rice and maize).

### 4.3. Phylogenetic Analysis and Subfamily Classification

Multiple sequence alignment (MSA) of the full-length protein sequences of *COL* was performed by MUSCLE (embedded in MEGA version 11.0.13) (default parameters) [85]. The phylogenetic relationship was constructed by the neighbor-joining method with the above alignment results (Poisson model, pairwise deletion, and other default parameters). The 1000 bootstrap replicates were taken to evaluate the reliability, and only those with bootstrap values higher than 50 were displayed. The subfamily delineation of *ApCOL* took the phylogenetic classification of *AtCOL* as a reference [9].

### 4.4. Sequence and Structural Analysis of COL Proteins

The online program ExPASy (https://web.expasy.org/protparam/ (accessed on 26 January 2024)) was utilized to predict the biochemical properties of ApCOL proteins, including amino acid numbers, molecular weights (MWs), and predicted isoelectric points (pIs), etc. [86]. Subcellular localization was performed by WoLF PSORT (https://wolfpsort.hgc.jp/ (accessed on 26 January 2024)), CELLO 2.5 (http://cello.life.nctu.edu.tw/ (accessed on 26 January 2024)), and Cell-PLoc (http://www.csbio.sjtu.edu.cn/bioinf/Cell-PLoc/ (accessed on 26 January 2024)) for multiple assessments [87,88,89]. The conserved motifs of ApCOL proteins were recognized via the Multiple Em for Motif Elicitation 5.4.1 (MEME, https://meme-suite.org/meme/ (accessed on 12 March 2024)) [90]. Gene structure information was obtained from gene annotation files.

### 4.5. Analysis of Cis-Regulatory Elements and Transcription Factor Binding Sites

The 2000 bp sequences of the *ApCOL* genes upstream of the ATG start codon were extracted by Perl script. The Plant CARE database (http://bioinformatics.psb.ugent.be/webtools/plantcare/html/ (accessed on 15 March 2024)) was applied to predict cis-regulatory elements (CREs) [91].

### 4.6. Plant Materials and Treatment Methods

The seeds of *A. paniculata* were derived from the Key Laboratory of Plant Secondary Metabolism and Regulation, Zhejiang Sci-Tech University, Hangzhou, China. The seeds were soaked in 40 °C warm water with natural cooling overnight to interrupt seed dormancy. The seedling stage was conducted in the artificial climate chamber, growing to 4–6 leaves for transplanting in the natural environment. Rapid-growth period *A. paniculata* were re-transferred to the artificial climate chamber for incubation so that the rate of uptake and the growth condition could be guaranteed. *A. paniculata* was stressed with gradient solutions; salt treatments were used with the most commonly used concentrations of NaCl (0, 50, 100 mM), while hormone treatments were set according to existing studies with SA, 6-BA, and NAA concentrations of (0, 5, and 10 μM). [92,93,94]. It was treated with a hand-held nebulizer, spraying the solution to the leaves until the leaves were well-wetted. The bottoms of the potted plants were placed with water storage devices for solution absorption and water replenishment. The time of treatment was picked in the evening to reduce the influence of plant photosynthesis. Each treatment was repeated three times. To capture the dynamics of the stress response, fresh young leaves were collected on days 5 and 10, respectively, after treatment, immediately placed in liquid nitrogen, and then stored in a refrigerator at −80 °C until reused.

### 4.7. RNA Extraction and Construction of Gene Expression Profiles

Total RNA from *A. paniculata* leaves was extracted using the FastPure Plant Total RNA Isolation Kit (Vazyme, Nanjing, China). The quantity and quality of all RNA samples were determined by microspectrophotometer (Thermo Fisher, NanoDrop 2000, Waltham, MA, USA). cDNA was synthesized using the Reverse Transcription Kit, and the product was diluted and used as a template. The above processes were performed according to the manufacturer’s instructions. The qRT-PCR was carried out with a ChamQ Universal SYBR qPCR Master Mix (Vazyme, Q311, Nanjing, China) on the QuantStudio 6 Flex Real-Time PCR System (Thermo Fisher, Waltham, MA, USA). A minimum of three replicates were used for each gene. The UBC was treated as an internal reference gene, and all primers are listed in the Supplemental Table S6. The 2^−ΔΔCt^ method was adopted for outcome analysis [95], and gene expression profiles were visualized in TBtools version v2.142 as log_2_-fold change values of gene expression [96]. When the relative expressions of genes (treatment/control) were ≥2 or ≤0.5 compared to the control group, they were considered as significantly up- or downregulated.

### 4.8. Subcellular Localization Analysis

The CDS of *ApCOL08* and *ApCOL09* without stop codon were constructed into the pCAMBIA1300-eGFP vector and cut with Xbal and BamHI. The recombinant vectors (pCAMBIA1300-*ApCOL08* and pCAMBIA1300-*ApCOL09*) and the empty vector (pCAMBIA1300-eGFP) were then transformed into Agrobacterium GV3101 and injected into approximately five-week-old tobacco leaves. After incubation under dark conditions for 48 h, GFP fluorescence was observed by laser scanning confocal microscopy.

### 4.9. Heterologous Expression of Genes in Yeast

The recombinant plasmid *pYES2*-*ApCOL08* was obtained by cloning CDS of *ApCOL08* onto *pYES2* vector using EcoRI and XhoI sites. The yeast expression vectors *pYES2* no-load control and *pYES2*-*ApCOL08* were converted into *S. cerevisiae* INVSc1 by the lithium acetate conversion method [76]. Specifically, the INVSc1 yeast containing the plasmid was shaken overnight at 30 °C in liquid medium containing 2% glucose SD-U to OD600 = 0.4 and centrifuged at 1500× *g*; the supernatant was then removed, and the same volume of SG-U liquid medium containing 2% galactose was added; the bacteria were shaken at 30 °C overnight for 24 h to induce expression, centrifuged to remove the supernatant, resuspended with sterile water, and diluted (10^0^, 10^1^, 10^2^, 10^3^) to SG-U + 0 M NaCl, SG-U + 0.5 M NaCl, SG-U + 1.0 M NaCl, SG-U + 1.3 M NaCl, SG-U + 1.5 M NaCl, SG-U + 2.0 M NaCl plates, which were placed in a 30 °C incubator and observed after 4 days and 7 days, respectively, and finally, pictures were taken [97].

## 5. Conclusions

In this study, we identified nine *ApCOL* genes and performed a comprehensive genomic analysis of the *ApCOL* gene family in *A. paniculata*, focusing on their evolution, structure, expression profile, and potential functions. Our results suggest that the *COL* gene family may have important roles in plant growth and development and stress response in addition to flowering regulation. We found that *ApCOL* genes were specifically expressed in different tissues. In addition, some genes could be significantly induced by hormones and salt stress, and we identified *ApCOL08* as a possible key regulatory gene. Subsequently, cloning and expression analysis of *ApCOL08* in *S.cerevisiae* showed enhanced tolerance to salt stress in yeast, suggesting that it may be a promising candidate gene for further exploration of salt tolerance studies. Since the successful cases of genetic engineering for *A. paniculata* are very limited, the only documented case is the successful expression of VIGS (virus-induced gene silencing) through *Agrobacterium* infestation of *A. paniculata* leaves, and we are currently trying to follow this method, and we will add the salt stress treatment to the experiment in the future, so as to further validate the biological function of the candidate genes. In addition, based on the fact that salt stress affects diterpene lactones, which are active components in *A. paniculata*, a salt-tolerant *A. paniculata* can be cultivated for planting in saline soils in the future, thus expanding the distribution of *A. paniculata* germplasm resources. In conclusion, this study not only contributes to the understanding of the evolutionary dynamics of *COL* genes, but also provides a new approach to improve salt stress tolerance in plant breeding.

## Figures and Tables

**Table 1:** Detailed information of nine COL proteins identified in *Andrographis paniculata*.

Name	Gene ID	Chromosome	Strand	Gene Length (bp)	Protein			Predicted Subcellular Localization
					Length (aa)	MW (kDa)	pI	
*ApCOL01*	CXN00019358	Chr1	+	1327	264	39.13744	5.5	Nucleus
*ApCOL02*	CXN00016323	Chr1	+	2228	247	43.25575	5.1	Chloroplast
*ApCOL03*	CXN00002171	Chr2	+	5067	326	35.31866	5.84	Nucleus
*ApCOL04*	CXN00012269	Chr2	−	4173	260	46.36184	6.24	Nucleus
*ApCOL05*	CXN00007803	Chr2	+	4053	204	47.51274	5.1	Nucleus
*ApCOL06*	CXN00005866	Chr13	−	1355	352	36.45549	5.2	Nucleus
*ApCOL07*	CXN00017383	Chr15	−	1212	207	41.87428	6.81	Chloroplast
*ApCOL08*	CXN00004299	Chr19	−	1536	211	48.41006	5.42	Nucleus
*ApCOL09*	CXN00005183	Chr21	+	1625	238	40.98486	5.95	Nucleus

MW, molecular weight; pI, isoelectric point.

## Data Availability

Data is contained within the article and Supplementary Materials.

## Supplementary Materials

The supporting information can be downloaded at https://www.mdpi.com/article/10.3390/ijms26020724/s1.
