# Supplementary material for: Genome-Wide Identification of CONSTANS-like (COL) Gene Family and the Potential Function of ApCOL08 Under Salt Stress in Andrographis paniculata
Source: Int J Mol Sci. 2025 Jan 16;26(2):724. doi: 10.3390/ijms26020724 (PMC11765704; doi:10.3390/ijms26020724)
Supplement: Supplementary file 1 [file ijms-26-00724-s001.zip › ijms-3404078-SM figures.docx]

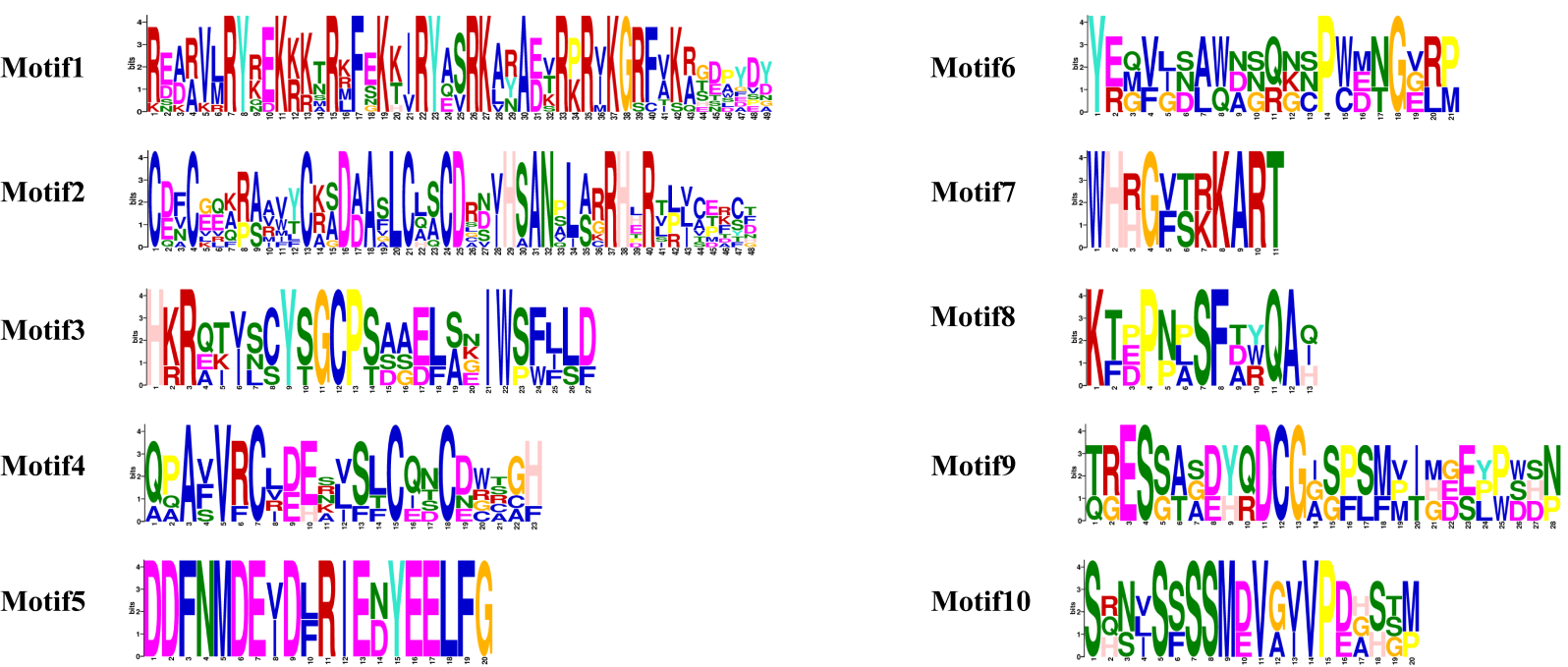


**Fig. S1.** Web logos of ten conserved motifs in *ApCOL* genes.


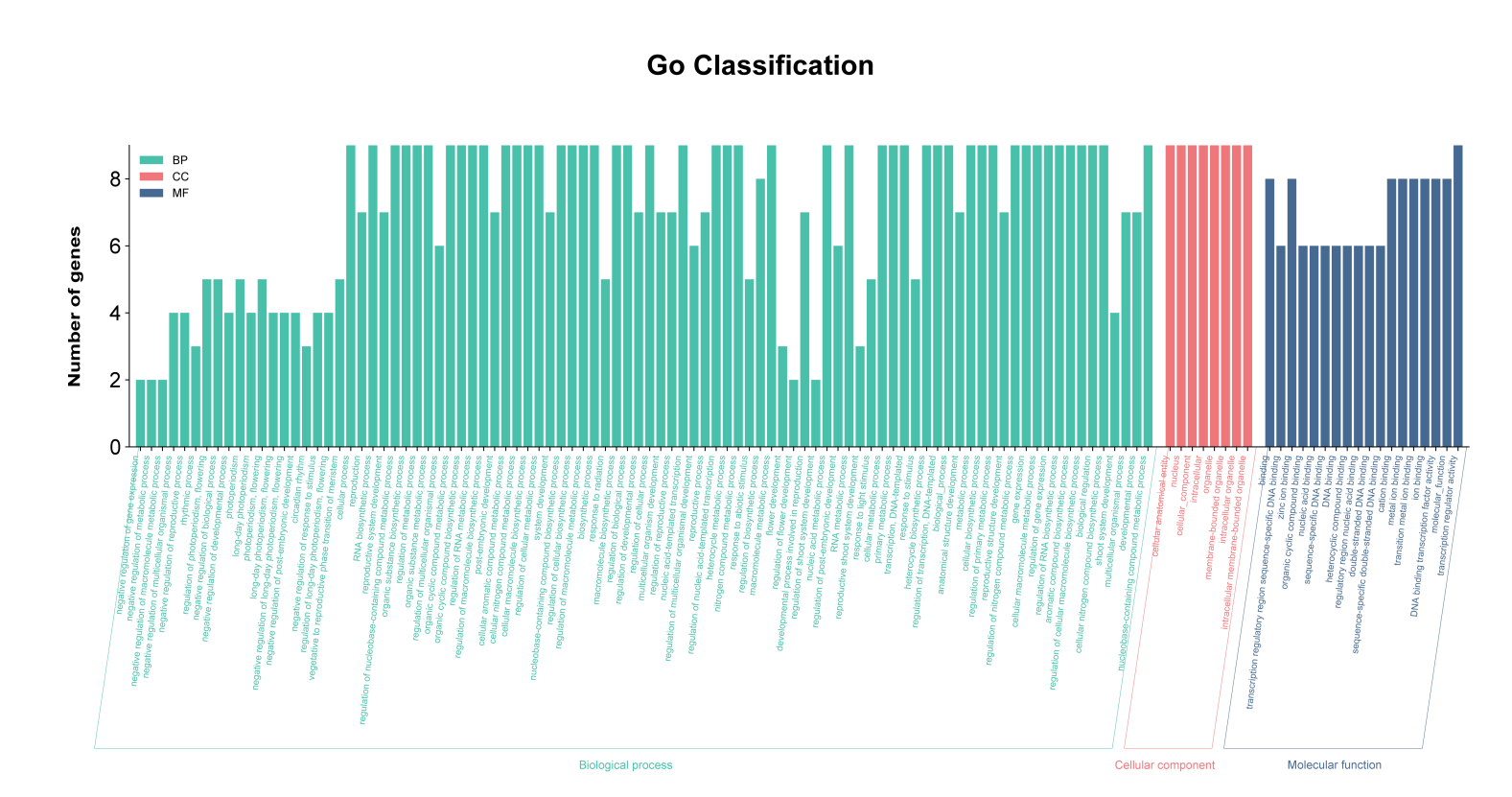


**Fig. S2.** GO annotation of *ApCOL* genes in *A. paniculata*.
